# Editorial: Role of Protein-Protein Interactions in Metabolism: Genetics, Structure, Function

**DOI:** 10.3389/fphar.2017.00881

**Published:** 2017-11-27

**Authors:** Amit V. Pandey, Colin J. Henderson, Yuji Ishii, Michel Kranendonk, Wayne L. Backes, Ulrich M. Zanger

**Affiliations:** ^1^Pediatric Endocrinology, Diabetology and Metabolism, University Children's Hospital Bern, Bern, Switzerland; ^2^Department of Biomedical Research, University of Bern, Bern, Switzerland; ^3^Division of Cancer Research, Jacqui Wood Cancer Centre, School of Medicine, University of Dundee, Dundee, United Kingdom; ^4^Laboratory of Molecular Life Sciences, Graduate School of Pharmaceutical Sciences, Kyushu University, Fukuoka, Japan; ^5^Center for Toxicogenomics and Human Health (ToxOmics), Genetics, Oncology and Human Toxicology, Universidade Nova de Lisboa, Lisbon, Portugal; ^6^Department of Pharmacology and Experimental Therapeutics, Louisiana State University Health Sciences Center New Orleans, New Orleans, LA, United States; ^7^Department of Molecular and Cell Biology, Dr. Margarete Fischer-Bosch-Institute of Clinical Pharmacology, Stuttgart, Germany; ^8^Eberhard Karls University of Tübingen, Tübingen, Germany

**Keywords:** cytochrome P450, POR, UGT, PXR, drug metabolism

This editorial describes the articles published under our research topic “Role of protein-protein interactions in metabolism: Genetics, structure, function.” Our aim was to bring together researchers working on drug, steroid, and xenobiotic metabolism with interest in protein-protein interaction for presenting their latest findings and share their opinions on recent advances in the field. Recent advances in genetics (Meyer, 2004) and structural biology have greatly enhanced our understanding of molecular details of diversity and differences behind control of metabolic processes. The topic attracted a wide range of manuscripts using genetics, proteomics, biochemical, and structural biological approaches in study of protein-protein interactions.

In six original articles, four reviews, and one mini-review, leading experts in the field described different approaches and use of advanced technologies in the study of protein-protein interactions related to metabolic processes.

In a review of human UDP-glucuronosyltransfares (UGTs) Fujiwara et al. discussed the current understanding of the structure and function of UGTs in relation to protein-protein interactions and oligomerization and summarized their own as well as other related studies on interactions of UGTs with other proteins (Fujiwara et al., 2010). Nakamura et al. described the modification of UGT2B3 by creation of an N-glycosylation site to alter its sensitivity toward CYP3A1. It has been known for some time that CYP3A4 can change the activities of UGTs in isoform specific manner (Ishii et al., 2014). Rouleau et al. used a proteomics approach to study the protein-protein interactions of human UGT1As with other proteins including UGTs, transporters, and dehydrogenases. Role of UGTs in gene regulation as well as metabolic regulation by interactions with other proteins has become an emerging area of interest (Audet-Delage et al., 2017).

Pavek provided a review of pregnane X receptor (PXR) interactions with other nuclear receptors and its role in gene regulation (Rulcova et al., 2010). Ryu et al. have reviewed the interactions of cytochrome P450 proteins (Omura, 2010; Zanger and Schwab, 2013) with the membrane associated progesterone receptors (MAPR). Many MAPRs share similarities to cytochrome b5 and therefore are evolutionary adapted for interactions with cytochrome P450s (Xie et al., 2011).

Udhane et al. explored the role of genetic variations in human NADPH cytochrome P450 oxidoreductase (POR) found in apparently normal human population, in the metabolism of drugs and steroid hormones. Human POR (Pandey and Flück, 2013) is a diflavin reductase containing both the flavin mononucleotide (FMN) and flavin adenine dinucleotide (FAD) co-factors in separate domains that are linked by a hinge segment and interacts with cytochrome P450 proteins (Zanger and Schwab, 2013) and other redox partners (Pandey and Flück, 2013; Riddick et al., 2013). Using CYP19A1 (aromatase) (Pandey et al., 2007) for steroid metabolism and CYP3A4 for drug metabolism (Flück et al., 2010), Udhane et al. found variable effects of POR genetic variants in the FMN binding and hinge regions of POR on the activities of CYP11A1 and CYP3A4. Režen et al. studied the polymorphisms of *CYP51A1* (Lewinska et al., 2013) for impact on interactions with POR. Using computational models Režen et al. predicted that *CYP51A1* variants R277L and D152G have lower binding affinity for POR.

A thorough review of the biophysical techniques used in study of Cytochrome P450 proteins as well their interactions with other proteins involved in xenobiotic metabolism was provided by Lampe. By examining the X-ray crystal structures of P450 enzymes, Reed and Backes were able to identify potential contact points for the formation of P450-P450 complexes when interacting in membranes. This information allows for the predictions of how P450 system proteins are organized in the endoplasmic reticulum as well as the functional consequences of these interactions (Davydov et al., 2013).

Campelo et al. performed a study on the salt-induced changes of the dynamics properties of human POR (also described as CPR, CYPOR) by changing specific amino acids of the hinge segment which were postulated to play a critical role in electron transfer to its redox partners. Striking changes in the salt-profile of cytochrome c reduction by POR were observed with several of mutations created by Campelo et al. These results demonstrated that both electrostatics and flexibility of the hinge segment in POR are critical. Knowledge on the molecular mechanism of POR's gated electron transfer is of importance for the understanding the crucial role POR's for the activity of its redox-partners. Such knowledge may shed light on the impact of specific human polymorphic variants of POR (Sim et al., 2009) in specific pathologies but may also find biotechnological applications, such as P450 mediated metabolite production (Bernhardt and Urlacher, 2014). Degregorio et al. applied a chimeric approach for finding the optimal redox conditions to support cytochrome P450s reactions. A chimeric protein consisting of the reductase domain of bacterium *Bacillus megaterium* BM3 and a modified CYP3A4 was created to achieve a P450 containing its own reductase domain for a stable and efficient electron transfer during catalytic reactions. By using different linkers in between reductase and P450, Degregorio et al. could achieve 2 to 3-fold maximum velocity and coupling efficiency compared to use of separate P450 and redox partner proteins (Munro et al., 1996).

In conclusion, this research topic illustrated the up-to-date status of the field by leading scientists and provided a current state of the art on the importance of protein-protein interactions in metabolism and their role in a range of human diseases as well as biotechnological applications of the findings obtained from basic studies. We hope that the information gained from publication of this research topic will stimulate research on the role of protein-protein interactions in metabolism and facilitate further advances in the field.

## Author contributions

All authors listed have made a substantial, direct and intellectual contribution to the work, and approved it for publication.

## Conflict of interest statement

The authors declare that the research was conducted in the absence of any commercial or financial relationships that could be construed as a potential conflict of interest.
